# Polyunsaturated Fatty Acids Modify the Gating of Kv Channels

**DOI:** 10.3389/fphar.2012.00163

**Published:** 2012-09-10

**Authors:** Cristina Moreno, Alvaro Macias, Angela Prieto, Alicia De La Cruz, Carmen Valenzuela

**Affiliations:** ^1^Instituto de Investigaciones Biomédicas “Alberto Sols,” Consejo Superior de Investigaciones Científicas-Universidad Autónoma de MadridMadrid, Spain

**Keywords:** *n*−3 PUFAs, Kv1.5, Kv11.1, gating, voltage-sensor

## Abstract

Polyunsaturated fatty acids (PUFAs) have been reported to exhibit antiarrhythmic properties, which are attributed to their capability to modulate ion channels. This PUFAs ability has been reported to be due to their effects on the gating properties of ion channels. In the present review, we will focus on the role of PUFAs on the gating of two Kv channels, Kv1.5 and Kv11.1. Kv1.5 channels are blocked by *n*−3 PUFAs of marine [docosahexaenoic acid (DHA) and eicosapentaenoic acid] and plant origin (alpha-linolenic acid, ALA) at physiological concentrations. The blockade of Kv1.5 channels by PUFAs steeply increased in the range of membrane potentials coinciding with those of Kv1.5 channel activation, suggesting that PUFAs-channel binding may derive a significant fraction of its voltage sensitivity through the coupling to channel gating. A similar shift in the activation voltage was noted for the effects of *n*–6 arachidonic acid (AA) and DHA on Kv1.1, Kv1.2, and Kv11.1 channels. PUFAs-Kv1.5 channel interaction is time-dependent, producing a fast decay of the current upon depolarization. Thus, Kv1.5 channel opening is a prerequisite for the PUFA-channel interaction. Similar to the Kv1.5 channels, the blockade of Kv11.1 channels by AA and DHA steeply increased in the range of membrane potentials that coincided with the range of Kv11.1 channel activation, suggesting that the PUFAs-Kv channel interactions are also coupled to channel gating. Furthermore, AA regulates the inactivation process in other Kv channels, introducing a fast voltage-dependent inactivation in non-inactivating Kv channels. These results have been explained within the framework that AA closes voltage-dependent potassium channels by inducing conformational changes in the selectivity filter, suggesting that Kv channel gating is lipid dependent.

## Introduction

The polyunsaturated fatty acids (PUFAs) present in nature belong to two main classes: the *n*−6 class and *n*−3 class. Both *n*−3 and *n*−6 PUFAs are essential nutrients that must be acquired from diet and are required for normal development and cellular function. In addition, mammals cannot convert *n*−6 into *n*−3 PUFAs. Certain vegetables can be a source for *n*−3 PUFAs, such as α-linolenic acid (ALA; 18:3 *n*−3), whereas other *n*−3 PUFAs, including eicosapentaenoic acid (EPA; 20:5 *n*−3) and docosahexaenoic acid (DHA; 22:6 *n*−3), can be obtained from marine sources (Holman, 1998). ALA is found in some vegetable oils (i.e., flaxseed, canola, and soybean) and walnuts, whereas marine *n*−3 PUFAs are found in fish and seafood. ALA is the precursor of EPA and DHA, but the conversion is limited and inefficient in organisms (Brenna, 2002). After ingestion, *n*−3 PUFAs are widely distributed to the cells and have effects on the membrane composition and function, eicosanoid synthesis, signaling, and the regulation of gene expression (Jump, 2002). A high dietary intake of *n*−3 PUFAs has also been linked to favorable physiological alterations, including reductions in the triglyceride levels, antithrombotic effects, enhanced immune function, and antiarrhythmic actions, which together may contribute to a lower risk of cardiovascular disease (Calder, 2004).

The dramatic increase in the *n*−6/*n*−3 ratio in the diet of the Western population after the industrial revolution has contributed to the rise in cardiovascular disease (Sinclair, 1953, 1956; De Caterina et al., 2003; Leaf et al., 2003). Moreover, there is a growing body of evidence that dietary *n*−3 PUFAs have an important role in the prevention of coronary heart disease by decreasing the risk of sudden cardiac death and, in particular, in preventing fatal ventricular arrhythmias (Burr et al., 1989; de Lorgeril et al., 1994; Singh et al., 1997; GISSI-Prevenzione Investigators, 1999; Albert et al., 2002; Leaf et al., 2003; Tanaka et al., 2008; Tavazzi et al., 2008). Finally, *n*−3 and *n*−6 PUFAs are metabolized to lipoxins, resolvins, and maresins (Serhan et al., 2008; Bannenberg and Serhan, 2010). These EPA- and DHA-derived lipid mediators (collectively termed Specific Pro-resolving Mediators; SPMs) are active, as anti-inflammatory agents, at very low concentrations (picomolar to nanomolar range), and are degraded locally by specific inactivating enzymes, characteristic for the bioactivity of autacoids. These SPMs can also slow the progression of diabetic onset, cardiovascular disease, and aging-associated pathologies through the regulation of innate and adaptive immune responses. The antiarrhythmic properties of *n*−3 PUFAs have been attributed to their ability to modulate the ion channels involved in the genesis and/or maintenance of cardiac action potentials. PUFAs inhibit the *I*_Na_, *I*_Kur_, *I*_TO, I__Kr_, and *I*_Ca_ voltage-gated ion channels and increase *I*_Ks_ (Honoré et al., 1994; Xiao et al., 1995, 1997; Doolan et al., 2002; Jude et al., 2003; Guizy et al., 2005, 2008; Verkerk et al., 2006; Dujardin et al., 2008), all of which are critical for biological signaling and regulating ion flux on a millisecond time scale.

In addition to their effects on the magnitude of the ionic currents, it has been demonstrated that membrane lipids can convert the Kv A-type channels into delayed rectifiers and vice versa. Within this framework, phosphoinositides (PIPs) remove the N-type inactivation, whereas AA converts the Kv delayed rectifiers into A-type rectifier channels (Oliver et al., 2004). Moreover, PIP_2_ modulates ion channel gating and the interaction between the Kv channels and β subunits (Loussouarn et al., 2003; Decher et al., 2008; Rodriguez et al., 2010; David et al., 2012). In the present review, we will focus on the effects of PUFAs on the gating of Kv1.5 and Kv11.1, the cloned counterparts of *I*_Kur_ and *I*_Kr_, respectively.

## Effects of n−3 PUFAs on the Gating of Kv1.5 Channels

Kv1.5 channels consist of four subunits, each containing six transmembrane segments (S1–S6). The voltage sensor of the channel is formed by the S1–S4 segments, and the segments S5 and S6, together with the P-loop form the ion conduction pathway. In the human heart, these subunits are expressed mainly in the atria (Roberds and Tamkun, 1991; Tamkun et al., 1991; Fedida et al., 1993). To sense changes in the membrane voltage, each ion channel is equipped with four voltage-sensor domains (S1–S4) connected to a central ion-conducting pore domain. It has been described that the bilayer-forming lipids interact with the S3 and S4 helices more strongly than with the S1 and S2 segments. Indeed, there are several reports indicating an important role of the lipid bilayer in the channel voltage-sensor (Butterwick and MacKinnon, 2010).

Regarding the pathophysiological roles of these channels, *I*_Kur_ and *I*_TO_ comprise the main human atrial repolarizing current. Moreover, *I*_Kur_ has not been recorded in the human ventricle. During chronic atrial fibrillation, there is an electrical remodeling that comprises shortening of the action potential duration and a lack of adaptation of the action potential duration to an accelerated heartbeat (Dobrev and Ravens, 2003; Wettwer et al., 2004). Because the Kv1.5 channels are mostly expressed in the atria, they have been proposed as pharmacological targets for antiarrhythmic drugs that are effective on supraventricular arrhythmias, such as atrial fibrillation (Varro et al., 2004; Wettwer, 2007; Dobrev et al., 2012). In fact, it has been proposed that the blockade of *I*_TO_ and/or *I*_Kur_ prolongs the atrial action potential at the plateau but not at the terminal phase of repolarization, leading to rotor termination (Pandit et al., 2005; Decher et al., 2006; Dobrev and Nattel, 2010).

The first study dealing with the effects of PUFAs on Kv1.5 channels was performed by Honoré et al. (1994) who analyzed two different PUFAs, AA and DHA. These authors reported the AA- and DHA-induced time-dependent blockade of these channels, the existence of an external binding site for *n*−3 PUFAs and also a change in the gating of Kv1.5 channels that appeared as a negative shift in the activation curve.

**Figure 1:**
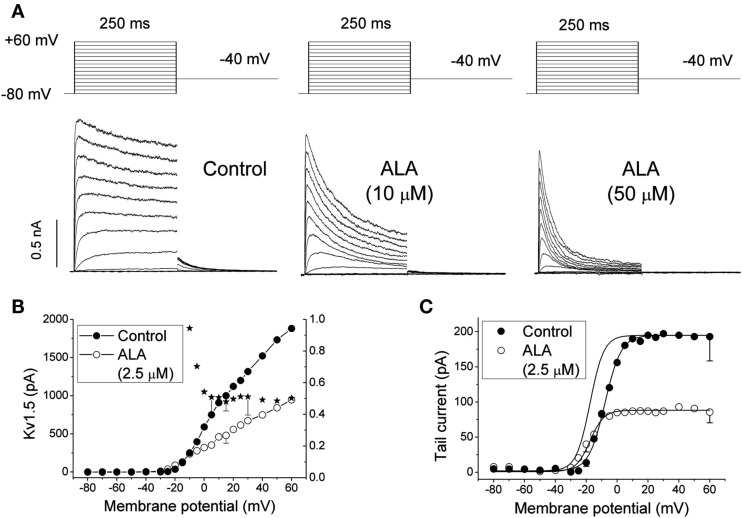
**(A)** Original traces of Kv1.5 channels obtained after applying the pulse protocol shown in the upper part of the figure in the absence (Control) and in the presence of 10 and 50 μM α-linolenic acid (ALA). **(B)** Current-voltage (IV) relationship of Kv1.5 in the absence and in the presence of 2.5 μM ALA. The plot also shows the mean relative current (*I*_ALA/I__Control_) at each membrane potential. **(C)** Kv1.5 activation curves obtained in the absence and in the presence of 2.5 μM ALA. The dotted line shows the activation curve obtained in the presence of ALA normalized to the matching control values. The data were obtained from Guizy et al. (2008).

The effects of ALA were qualitatively similar to those previously reported for DHA and AA (Honoré et al., 1994; Guizy et al., 2008). This study also showed that ALA both decreases the magnitude of the Kv1.5 current in a time-dependent manner and also modifies the gating of the channel (Figure 1). In fact, ALA shifts the activation curve toward negative membrane potentials, and an acceleration of the activation kinetics was observed in the range of the activation of the channels, a result that was likely due to the negative voltage shift (Guizy et al., 2008). A similar shift in the activation voltage was reported for the effects of AA and DHA on the Kv1.1 (Gubitosi-Klug and Gross, 1996), Kv1.2, and Kv11.1 channels (Poling et al., 1995; Guizy et al., 2005). Similar to what was previously described for AA and DHA, the blocking effects of ALA on the Kv1.5 channels only appeared when the PUFA was added from the external side of the membrane. In contrast, when it was applied from the inner side of the cell membrane, the magnitude of the current was not modified, but a hyperpolarizing shift of the activation curve and a slower deactivation time course of the current were observed (Figure 1). This finding indicated that the blocking effects produced by ALA on the Kv1.5 channels were due to the interaction with an external domain of the channel or, alternatively, an external domain of another molecule interacting with the ion channel. However, ALA modified the gating of the channel when it was applied from both sides of the membrane. Block of the Kv1.5 channels induced by ALA increased in the range of the membrane potentials that coincided with those of Kv1.5 channel activation, suggesting that the ALA-Kv1.5 interaction is voltage-dependent and likely coupled to the channel gating.

The flanking S5 and S6 segments are considered to contribute to the presumably wider intracellular mouth of the ion channel (Aiyar et al., 1994; Lopez et al., 1994) and contain binding sites for quaternary ammonium open-channel blockers and similarly acting drugs, such as quinidine or bupivacaine (Choi et al., 1993; Valenzuela et al., 1995, 1996; Yeola et al., 1996; Arias et al., 2007), and the Kvβ1.3 regulatory subunit (Decher et al., 2008; David et al., 2012). Block of Kv1.5 channels by *n*−3 and *n*−6 PUFAs resembles the effects of open-channel blockers and Kvβ1.3 regulatory subunits (Snyders et al., 1992; Valenzuela et al., 1995; Yeola et al., 1996; Franqueza et al., 1997; Gonzalez et al., 2002b; Decher et al., 2005, 2008; Arias et al., 2007; David et al., 2012). However, although these open-channel blockers interact with the inner part of the ion pore (Yeola et al., 1996; Franqueza et al., 1997; Decher et al., 2004), the PUFAs blockade appeared to be the consequence of their interaction with an external binding site in the channel. This issue concerning to the action mechanisms by which PUFAs modify ion channel activity has been a matter of debate. In fact, there are several pieces of evidence suggesting direct effects on ion channels (Xiao et al., 2000, 2001), whereas others reports claim that the effects of PUFAs represent the consequence of their effects on the physical properties of the lipid bilayer (Girshman et al., 1997; Bruno et al., 2007; Lundbaek, 2008). In fact, the *n*−3 and *n*−6 PUFAs are amphiphilic substances that may modulate the ion channel function by one of the following mechanisms: (a) changing the biophysical characteristics of the lipid bilayer; (b) modifying the hydrophobic interactions between the channel protein and the lipid bilayer, or (c) interacting specifically with an amino acid involved in the gating or the permeation of the channel (Oliver et al., 2004; Boland and Drzewiecki, 2008; Lundbaek, 2008; Meves, 2008). Recently, Decher et al. (2010) performed a study to elucidate the mechanism by which PUFAs modify the Kv1.x gating and the effects of AA and DHA on “edited” Kv1.x channels. Kv1.1 belongs to the increasing number of proteins having an amino acid sequence that is altered by RNA editing (Hoopengardner et al., 2003). In the “edited” Kv1.1 channels, an isoleucine facing the inner cavity of the pore is replaced by valine (I400V). This study shows that the effects of AA or other PUFAs (such as DHA) were strongly reduced in the “edited” Kv1.1 channels and also that, in heteromeric channels, the presence of only one “edited” Kv1.1 subunit was sufficient to decrease the affinity of PUFAs (Decher et al., 2010). This study concludes that the endogenous lipids AA and DHA produce an open-channel block of Kv channels, mimicking an increase in the rate of inactivation. These lipid effects are direct and can be antagonized by RNA editing of the Kv1.1 channels. However, the existence of “edited” Kv1.5 channels has not been demonstrated in the heart, brain, or other tissues. It is known that the isoleucine at position 400 is highly conserved in the Kv1.x channels and is located at the middle of the S6 helix, forming part of the wall of the central cavity of the ion channel (Long et al., 2005, 2007). In Kv1.5, the equivalent I400V position corresponds to I508, and the blocking effects of AA were also decreased in Kv1.5 I508A mutant channels. When the Kv1.1 channels were co-expressed with Kvβ1.1, the fractional block of the steady-state current by AA was strongly reduced (Decher et al., 2010). These data are consistent with the idea that Kvβ1.1 and AA compete for the same or overlapping binding sites located in the inner pore cavity, similar to the mode of action of quinidine and bupivacaine on Kvβ1.3 (Arias et al., 2007). Thus, the molecular mechanism of inactivation by AA might be analogous to the N-type inactivation by the Kvβ1 subunits (Decher et al., 2010). These results strongly suggest that, in contrast to the external binding site proposed by other authors (Honoré et al., 1994; Guizy et al., 2008), PUFAs seem to bind to an internal binding site that overlaps with the Kvβ1.3 and bupivacaine-binding site (Arias et al., 2007; Decher et al., 2008, 2010).

Why do PUFAs induce Kv1.5 block from the external side of the cell membrane? A possible explanation might be that when externally applied, PUFAs rapidly cross the lipid bilayer and bind to their internal receptor in the ion channel (Decher et al., 2010). Moreover, is it possible that no blocking effects are observed when PUFAs are applied from the internal side of the membrane. We may explain this unexpected behavior on the basis that, under these experimental conditions, PUFAs diffuse through the cell membrane into the bath faster than from the pipette to the membrane, as has been reported for other agents (DeCoursey, 1995; Brock et al., 2001; Macias et al., 2010).

## Effects of PUFAs on the Gating of Kv11.1 Channels

Kv11.1 channels are encoded by the human ether-à-go-go-related gene (KCNH2), and the ionic current generated after their activation is responsible for the rapid delayed rectifier potassium current (*I*_Kr_) in the heart and several other cell types. These channels are homotetramers of a protein formed by six membrane-spanning domains with intracellular N- and C-termini. *I*_Kr_ is characterized by a rapid activation at −30 mV and a strong inward rectification at positive potentials, which is due to the rapid voltage-dependent C-type inactivation. Inward rectification results from the fact that channel inactivation develops faster than channel activation at positive potentials and limits the amount of time that channels exist in the open state (Smith et al., 1996; Spector et al., 1996).

The KCNH2 gene has been identified as the locus of mutations associated with type 2 long QT syndrome (LQTS2; Splawski et al., 2000; Roden et al., 2002; Kass and Moss, 2003). LQTS is a complex disease characterized by a marked QT interval prolongation and polymorphic ventricular tachycardia called torsades de pointes (TdP), causing syncope, seizures, and sudden death (Splawski et al., 2000; Roden et al., 2002; Kass and Moss, 2003; Shimizu, 2005). More than 290 mutations in the KCNH2 gene have been described, including frameshifts, insertions, deletions, and missense and nonsense mutations (Splawski et al., 2000; Roden et al., 2002; Kass and Moss, 2003; Shimizu, 2005). Mutant Kv11.1 channels producing LQTS2 generate reduced outward potassium currents that can be due to (a) the generation of non-functional channels, (b) altered channel gating, and/or (c) abnormal protein membrane trafficking (Thomas et al., 2003; Shimizu, 2005).

There is only one study in which the effects of acute PUFAs were studied in Kv11.1 channels (Guizy et al., 2005), reporting that the effects of AA and DHA block the Kv11.1 channels in a manner consistent with an open-channel block mechanism. The blockade induced by both of these PUFAs steeply increased in the range of the membrane potentials coinciding with the range of Kv11.1 channel activation, suggesting (similar to that reported for the Kv1.5 channels) that their binding may derive a significant fraction of its voltage sensitivity by coupling to the channel gating. Unfortunately, at strong depolarizing voltages, the open and inactivated conformations of the Kv11.1 channels are in rapid equilibrium, making it difficult to unequivocally identify the state(s) with which these two PUFAs interact.

**Figure 2:**
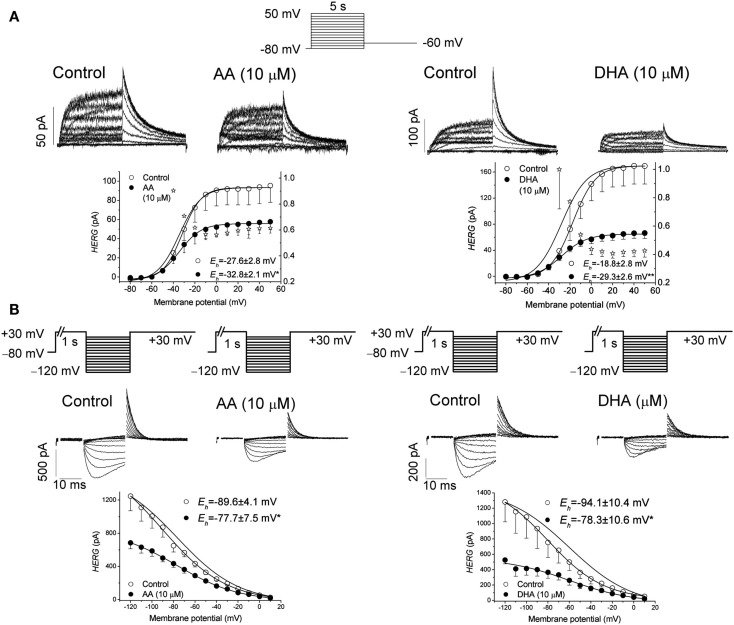
**(A)** Original traces of Kv11.1 channels obtained after applying the pulse protocol shown in the upper part of the figure in the absence (Control) and in the presence of arachidonic acid (AA) and docohexaenoic acid (DHA) at 10 μM. This panel also shows the activation curves of the Kv11.1 channels in the absence and in the presence of AA and DHA. **(B)** Apparent voltage dependence of channel availability. The pulse protocol used to obtain each data point is shown at the top. Original traces obtained after applying such a pulse protocol in the absence and in the presence of 10 μM AA and DHA. Corrected data for the deactivation together with the Boltzmann fit. The data were obtained from Guizy et al. (2005).

Although AA induced a similar inhibition of the Kv11.1 current when measured at the end of depolarizing pulses and at the maximum tail currents, DHA inhibited this current to a higher extent when measured at the maximum tail current, suggesting an open-channel interaction mechanism (Figure 2). During depolarization, the Kv11.1 channels inactivate faster than they activate, and, thus, the amplitude of the current is reduced. Upon repolarization, the closed channels recover from inactivation with a very fast kinetics, resulting in tail currents with higher amplitudes than the maximum activated current (Smith et al., 1996; Spector et al., 1996). Experiments in which the transition from the closed to the inactivated state was removed demonstrated that both AA and DHA bind to the open state of the Kv11.1 channels in a time- and use-dependent manner, as previously described for cocaine and bupivacaine-type local anesthetics (Zhang et al., 2001; Gonzalez et al., 2002a). In agreement with these results, the blockade of Kv11.1 channels induced by AA and DHA measured after the recovery from fast inactivation was similar (for AA) or higher (for DHA) than that measured after a long depolarizing pulse in which most of the Kv11.1 channels were in the inactivated state (Snyders and Chaudhary, 1996; Spector et al., 1996; Guizy et al., 2005).

The time-dependent interaction with the ion channel was also evident in the deactivation process of the Kv11.1 channels that was accelerated in the presence of both AA and DHA. However, AA and DHA did not modify the onset kinetics of the inactivation process or the recovery process (Guizy et al., 2005). The faster deactivation induced by both of the PUFAs, together with their lack of effect on the recovery kinetics, are consistent with an open-channel block mechanism, as has been proposed for propafenone (Arias et al., 2003). Therefore, the time-, use-, and voltage-dependent interaction with Kv11.1 channels suggest that both AA and DHA preferentially bind to the open state of these channels and that DHA exhibits a higher affinity for this state of the channel (Guizy et al., 2005).

It was also reported that AA and DHA produced a positive shift of the inactivation curve, which was interpreted to be the consequence of PUFAs binding to a closed state of the channel. This interaction is likely to influence the apparent steady-state inactivation and maybe the activation process (Guizy et al., 2005). All of these results suggest that AA and DHA preferentially block the open state of Kv11.1 channels but also that they interact with a closed state, thus producing changes in the channel gating (Figure 2). However, the similar degree of AA-induced inhibition of the current at the end of long depolarizing pulses (when most channels are inactivated), at the maximum tail current or at the maximum peak current after the recovery of C-type inactivation cannot permit us to rule out an interaction between AA and the inactivated state of the Kv11.1 channels. Similarly, AA has also been reported to inhibit Kv11.1, Kv11.2, and Kv11.3 currents recorded from clonal somatomammotrophic GH_3_/B_6_ cells (Schledermann et al., 2001). These results show a decrease of the current and a marked acceleration of the deactivation. All of these results that involve a shift of the activation and inactivation curves, together with a modification of the deactivation kinetics, may suggest changes in the channel gating.

As stated above, it has been described that AA regulates the inactivation process in other potassium channels, introducing a fast voltage-dependent inactivation into non-inactivating Kv channels (Oliver et al., 2004). These results are in agreement with those reported by Guizy et al. (2005). Oliver et al. (2004) explain these results by proposing that AA inserts into the cell membrane from either side, interacts with the channel protein and allosterically induces a rapid closure of the open Kv channel pore through conformational modifications in the selectivity filter.

## Effects of PUFAs on Kv4 and Kv7.1 + KCNE1 Channels

As stated above, PUFAs are also able to modulate other cardiac ion channels present in the heart. Focusing on the outward K^+^ currents, it has been reported that DHA and AA block *I*_TO_, whereas DHA increases *I*_Ks_ (Singleton et al., 1999; Doolan et al., 2002; Boland et al., 2009; Xu et al., 2010). These studies show that both AA and DHA decrease the magnitude of Kv4.3 and also that AA shifts the activation curve, but not the inactivation curve, in the presence of KChiP1b but not in the absence of this accessory subunit. However, DHA shifts the activation curve both in the absence and in the presence of KChiP1b (Boland et al., 2009). In contrast, it has been reported that DHA increases *I*_Ks_ when Kv7.1 + KCNE1 was transfected into *Xenopus* oocytes (Doolan et al., 2002). Taking all of the effects of DHA on all of the outward potassium currents together, we can expect small changes in the duration of the cardiac action potential, as the blocking effects on *I*_Kr_, *I*_Kur_, *I*_TO_ can be counteracted by the effects on *I*_Ks_.

## Concluding Remarks

PUFAs have diverse effects on cardiac ion currents. All of the reports in which PUFA effects have been studied on Kv channels demonstrate that these molecules modify the channel gating, indicating that the gating of Kv channels can be modified by lipids. This is not a surprising result, as it has been previously described that the S3 and S4 helices of the voltage-sensor domain of KvAP channels exhibit specific interactions with phospholipids. Conversely, the S1 and S2 helices might present a generic hydrophobic surface that is more equally satisfied by detergents and long-chain lipids (Bond and Sansom, 2007; Butterwick and MacKinnon, 2010). It has also been described that a diet rich in PUFAs is able to modify the lipid bilayer composition. The effects of PUFAs can be different and even opposite when they are acutely or chronically applied. In fact, *n*−3 PUFAs decrease Kv1.5 channels under both experimental conditions (Guizy et al., 2008). However, PUFAs decreased the magnitude of the Kv11.1 current after an acute exposure, whereas they did not modify this potassium current recorded in myocytes obtained from animal fed with a diet rich in *n*−3 PUFAs (Verkerk et al., 2006; Den Ruijter et al., 2012). These apparent discrepant results may be explained by two different effects of PUFAs on ion channels: a direct effect on the ion channel and another produced after changing the membrane biophysical properties. Both mechanisms can modify the gating of the ion channel; however, the effects on the ion current magnitude will likely derive from direct PUFA-channel interactions. Further lipidomic and electrophysiological studies using mutant Kv11.1 channels are necessary for a better understanding of the effects of PUFAs on the gating of Kv channels.

## Conflict of Interest Statement

The authors declare that the research was conducted in the absence of any commercial or financial relationships that could be construed as a potential conflict of interest.
